# Editorial: Bridging the gap: implementing building blocks of the nervous system to simulate neuronal functions at different levels

**DOI:** 10.3389/fnint.2023.1335723

**Published:** 2023-11-29

**Authors:** Yasuhito Watanabe, Yutaka Sakaguchi, Janet L. Paluh

**Affiliations:** ^1^AI inside Inc., Tokyo, Japan; ^2^Department of Mechanical Engineering and Intelligent Systems, The University of Electro-Communications, Chofu, Tokyo, Japan; ^3^College of Nanotechnology, Science and Engineering, University at Albany, State University of New York, Albany, NY, United States

**Keywords:** building blocks of nervous system, interconnection, combinatory therapeutic intervention, motor neuron networks, motor disorders, affective pain, social behavior

Mechanisms underlying how the nervous system functions so exquisitely in relation to its cellular architecture, molecular signaling components, networking and learning continues to be extensively studied. Pathological conditions in neurological diseases give some insights into these mechanisms and networks however, such understanding is often a snapshot and does not necessarily explain fully the phenomena seen across different levels, including normal and perturbed cognition.

The nervous system's heterogeneous and complex structure must interconnect specific areas mapped to specific types of information processing including sensory, motor and executive functions. At the same time, common elements also exist in the system, such as neuron/non-neuronal glial cell types, developmental/aging processes, and structures of cellular compartments including axons, dendrites, and synapses all performing in a four dimensional space with time.

Whereas in human-driven microprocessor chip manufacturing that is a top-down approach, it is well-appreciated that biological systems function in a bottom-up scenario. Therefore, specific cognitive mechanisms might be better understood through evaluation of specific information inputs, their synaptic connections, firing patterns, gene expression, molecular localization, and their combinations. A multitude of studies, including omics studies and spatial RNASeq are being conducted at the level of components to whole system architecture and begin to characterize the nervous system in certain key aspects. There remains a great need to improve our understanding of the nervous system, particularly how different levels of biological processes are interconnected and specifically interacted to generate various brain functions. A better understanding of regional and multi-scale nervous system cooperative or interactive functions would contribute to the development of novel medicines and other related technologies. In this Research Topic, we sought to focus on such approaches and exploring new methodologies.

Specifically, the objectives of the Research Topic were to collect studies examining how different levels of biological phenomena influence other levels, thereby obtaining insights on a future blueprint of how various building blocks of the nervous system bridge different levels of phenomena.

Our Research Topic includes four articles that fit to our scope. These studies conducted biological experiments either *in vitro* or *in vivo* for simulating the biological process related to the neuropathological conditions in human and tested their hypotheses. Manipulation of oxidative levels, visual input, mechanoreceptor action, and folate metabolism were conducted and the phenomena at different levels observed, including motor neuron networks, gastrointestinal symptoms, motoric activity, affective pain, and social behavior. The correlation between different interactive and functional levels were tested by modifying the experimental conditions including adding interventions such as activity inhibition at specific time points relative to the event of the stress.

Fiskum et al. found that activity inhibition during hypoxia improves functional outcomes in motor neuron networks *in vitro*. In this study, they corroborated that outcomes of hypoxia can be influenced by motor neuron network activity *in vitro*, showing the connection of pathophysiological outcome with intervention at the level of neuronal activity.

Mao et al. reported that visual stimulation synchronized to rotation movement is linked to alleviation in gastrointestinal symptoms and motor disorders in rats. Here the connection of visceral pathophysiological outcome with a sensory input, a visual stimulation synchronized to movement through retina *in vivo* was corroborated.

Noble et al. showed that C-low threshold mechanoreceptor activation can trigger affective pain in spinal cord injured mice. In this study, affective pain was linked with the activity of neurons in the circuit, thereby demonstrating connection of the type-specific-neuronal activity in the specific circuit with pain outcomes.

Sadigurschi et al. demonstrated that genetic impairment of folate metabolism regulates cortical interneurons and social behavior. In this study social behavior was bridged with folate metabolism and the histological and compositional abnormalities caused by the genetic abnormality. Direct link of a histological abnormality and social behavioral abnormality has not been shown; however, linking of an abnormality in multiple levels was shown.

These studies demonstrate how different levels of biological activity influence neurophysiological outcomes including pathological phenomena, revealing how properties at one level influence the property of another level. Future studies with complementary computational reconstitution of these observed influences in outcome by intervention in other levels may further help our understanding of how the nervous systems work in different levels hand in hand from molecular to cognitive levels. This advance may bring us further versatile discoveries, such as new type of effective combinatory therapeutic intervention targeting the interconnecting building blocks of several biological levels.

## Author contributions

YW: Conceptualization, Investigation, Writing—original draft, Writing—review & editing. YS: Conceptualization, Investigation, Writing—review & editing. JP: Writing—review & editing.

